# Osmanthus Fragrans Loaded NIPAAM Hydrogel Promotes Osteogenic Differentiation of MC3T3-E1

**DOI:** 10.3390/gels8100659

**Published:** 2022-10-15

**Authors:** Bin Huang, Mengyao Zhao, Mingzhe Yang, Lu Rao, Chizhou Wu, Yuzhu Hu, Huangqin Chen, Yuesheng Li

**Affiliations:** 1 Department of Stomatology, School of Stomatology and Ophthalmology, Xianing Medical College, Hubei University of Science and Technology, Xianning 437100, China; 2 Hubei Key Laboratory of Radiation Chemistry and Functional Materials, Non-Power Nuclear Technology Collaborative Innovation Center, Hubei University of Science and Technology, Xianning 437100, China

**Keywords:** osmanthus fragrans-loaded NIPAAM, hydrogel, electron beam radiation, osteogenic differentiation, network pharmacology, molecular docking

## Abstract

There is an urgent need to find long-acting, natural osteogenesis-promoting drug systems. In this study, first the potential targets and mechanism of osmanthus fragrans (O. fragrans) extract in regulating osteogenic differentiation based on autophagy were analyzed by network pharmacology and molecular docking. Then, osmanthus fragrans was extracted using the ethanol reflux method and an osmanthus fragrans extract loaded Poly N-isopropylacrylamide (OF/NIPAAM) hydrogel was prepared by electron beam radiation. The chemical components of the osmanthus fragrans extract and the microstructure of OF/NIPAAM hydrogels were characterized by ultraviolet-visible spectrophotometry (UV-Vis) and X-ray diffraction (XRD), respectively. Mouse embryonic osteoblast precursor cells MC3T3-E1 were cultured with different concentrations of OF/NIPAAM hydrogel to discover cell proliferation activity by CCK-8 assay. Alkaline phosphatase (ALP) staining and alizarin red staining were used to observe the differentiation and calcification. Through experimental exploration, we found that a total of 11 targets were predicted, which are TP53, CASP3, SIRT1, etc., and osmanthus fragrans had good binding activity to TP53. In vitro, except for proliferation promotion, OF/NIPAAM hydrogel enhanced ALP activity and formation of mineralized nodules of MC3T3-E1 cells at a concentration equal to or less than 62.5 μg/mL (*p* < 0.05). The addition of autophagy inhibitor 3-methyladenine (3-MA) reduced ALP activity and mineralized nodule formation.

## 1. Introduction

Osmanthus fragrans (O. fragrans), variously known as fragrant olive or sweet olive, is an evergreen shrub and is widely cultivated in Asia, from the Himalayas to China, Korea, Japan, and Thailand, due to its attractive color and strong, apricot-like fragrance [1]. It is a traditional ornamental plant with more than 2500 years of cultivation history in China, and it was not introduced to Europe until the late 18th century [2]. More than 160 osmanthus fragrans cultivars have been identified based on phenotypes and classified into four groups, Albus group (Yingui group), Luteus group (Jingui group), Aurantiacus group (Dangui group), and Asiaticus group (Sijigui group), according to the flowing season and flower color [3].

In addition to their extremely high ornamental value, osmanthus fragrans flowers also have high economic value and are widely used as edible products and in traditional folk medicine. The Chinese ancients created several methods of consuming osmanthus fragrans flowers as an additive to improve the flavor of Chinese traditional food such as tea, honey, cake, jam, and wine [4]. The *Compendium of Materia Medica* mentioned that osmanthus fragrans flowers have cough-relieving and phlegm-reducing effects [5] and have been used as folk medicine in rheumatism and stomachache treatment for a long time [6]. With the increasing attention, more extensive research on osmanthus fragrans flowers has been carried out in terms of pharmacological activities. Osmanthus, for example, containing nutritional and bioactive compounds, has been described to strengthen immune ability, lower blood pressure, promote digestion, and prevent obesity and diabetes [1]. Osmanthus fragrans not only has the above-mentioned health care functions, but also has strong antioxidant and anti-inflammatory effects, and has great potential for pharmaceutical application. A clinical trial showed that daily consumption of osmanthus fragrans flowers beverage for 7 days could enhance the antioxidant levels in healthy people [7], though it exhibited significant decreased antioxidant activities during simulated digestion [8]. Among 51 edible and wild flowers from China, osmanthus fragrans showed the highest antioxidant capacity, with a FRAP value of 163.57 μmol Fe (II)/g [9]. The flowers were also found to have much higher antioxidant capacity among 30 common flowers, with FRAP, DPPH, and TEAC values of 182.57, 250.67, and 223.85 μmol Trolox/gDW, respectively [10]. It has been identified that more than 20 phytochemicals in osmanthus fragrans, such as acteoside [11], salidroside [12], and ligustroside [13], have antioxidant effects. Osmanthus fragrans contains a large number of antioxidant compounds and has strong free radical scavenging ability, which provides a new strategy for the treatment of oxidative-stress-related diseases.

Oxidative stress plays a critical role in bone homeostasis during the bone remodeling processes, including osteoblast-induced bone formation and osteoclast-induced bone resorption. The potential relationship between autophagy and oxidative stress in bone remodeling has been increasingly reported. A growing number of drugs appear to exert protective effects on bone homeostasis through regulating autophagy (which can alleviate oxidative stress) [14,15]. However, research on the role of osmanthus fragrans in osteogenesis is limited. Furthermore, whether regulation of osmanthus fragrans in the osteogenic process is related to autophagy has not yet been fully established, which encourages us to conduct further investigations.

With the improvement of living standards, people have begun to pursue a long-acting, reliable, and side-effect-free therapeutic agent, while conventional medical agents no longer meet the needs of the public. Therefore, research on new drug systems is imminent. Smart hydrogels have attracted great attention from researchers in the medical field due to their ability to respond to external stimuli such as electric fields, magnetic fields, temperature, light, and pH. Among them, the NIPAAM thermo-sensitive hydrogel has become a research hotspot [16]. The traditional hydrogel synthesis process needs to add an initiator and catalyst, which has the disadvantages of low purity of the polymer product, slow reaction process, low efficiency, and easy generation of pollutants during the synthesis process. Electron beam radiation technology bombards or irradiates objects with accelerated electron beams to cause physical, chemical, and biological reactions that are difficult to trigger by conventional methods. It has the advantages of simple synthesis method, no residue of related substances, and normal temperature action.

The osmanthus fragrans extract, with a variety of components, can exert synergistic effects through multiple targets and multiple pathways. It is difficult to analyze them systematically and comprehensively by traditional experimental methods. Network pharmacology is a systematic analysis method based on drug–disease-related target genes and protein interaction networks. Molecular docking is a theoretical simulation method that uses computer technology to explore the interaction between receptors and drug molecules and predict their binding mode and affinity. These two technologies can save costs for new drug development and make experiments more targeted.

Therefore, network pharmacology was used to predict the target genes, and the mechanism of osmanthus fragrans promoting osteogenic differentiation was analyzed by molecular docking technology. Furthermore, the osmanthus fragrans extract and OF/NIPAAM hydrogels were prepared successfully by the ethanol reflux method and electron beam radiation, respectively. Various scientific experiments such as CCK-8, alkaline phosphatase (ALP) staining, and alizarin red staining were used for validation. The present study may provide a novel insight for basic research and subsequent application of osmanthus fragrans in promoting bone formation.

## 2. Results

### 2.1. Screening of Potential Targets of Osmanthus Fragrans Extract

**Figure 1:**
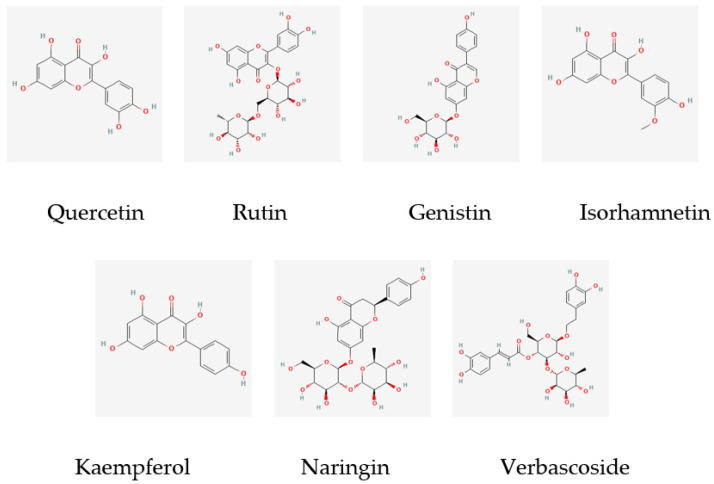
Molecular structure of 7 main components of osmanthus extract.

The main components of the osmanthus fragrans extract obtained through a literature search and data mining are the following seven kinds: quercetin, rutin, genistin, isorhamnetin, kaempferol, naringin, and verbascoside. The molecular structures are shown in Figure 1. A total of 275 potential targets of these 7 components were obtained through the SwissTarget Prediction database.

### 2.2. Identification of Overlapping Gene and Construction of the PPI Network

**Figure 2:**
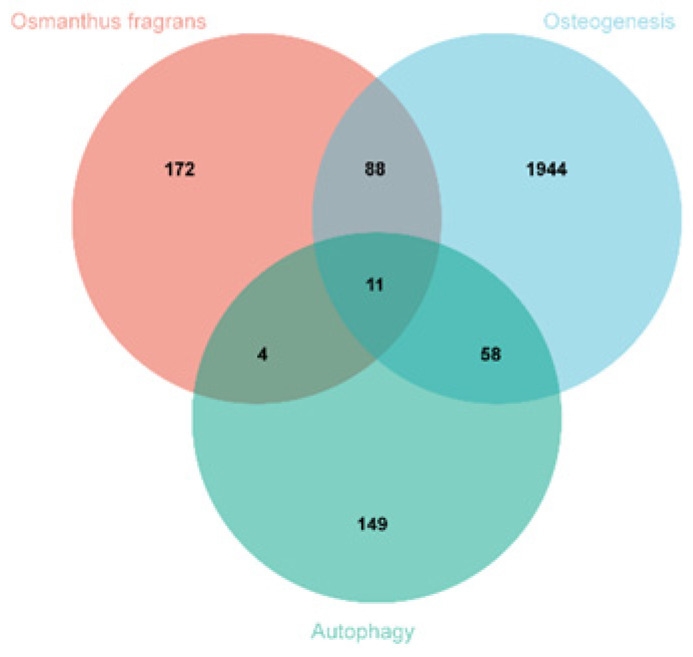
Identification of overlapping osmanthus fragrans, osteogenesis-, and autophagy-related genes.

**Figure 3:**
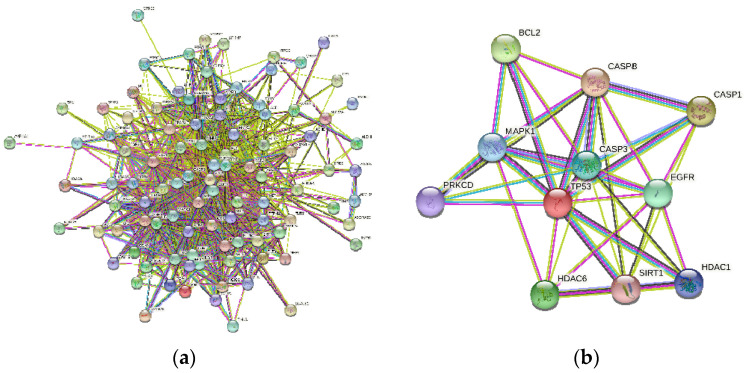
The PPI network analyzed visualized by Cytoscape software. (**a**) PPI network of 99 overlapping genes between osmanthus fragrans and osteogenesis-related genes. (**b**) PPI network of 11 overlapping genes of osmanthus fragrans, autophagy-, and osteogenesis-related genes.

A total of 275 osmanthus fragrans related genes, 2101 osteogenesis-related genes, and 222 autophagy-related genes were screened. The number of overlapping genes between osmanthus fragrans and osteogenesis-related genes was 99. The 11 overlapping genes (Figure 2) of these three were arranged in a sequence as follows: TP53, CASP3, SIRT1, HDAC6, MAPK1, CASP8, CASP1, BCL2, HDAC6, EGFR, and PRKCD. The PPI network of overlapping genes, as shown in Figure 3, was constructed by the STRING database and Cytoscape software. The PPI network maps have nodes and edges. Nodes represent proteins, and edges represent interactions between proteins. The higher the degree of network connectivity, the closer the relationship between proteins. Map A (99 overlapping genes) yielded 99 nodes and 990 edges, while map B (11 overlapping genes) yielded 11 nodes and 37 edges. All of these indicated that the osmanthus fragrans extract promotes osteogenesis based on the multi-component, multi-gene, and multi-target synergistic effects.

### 2.3. GO and KEGG Enrichment Analysis of Overlapping Genes

**Figure 4:**
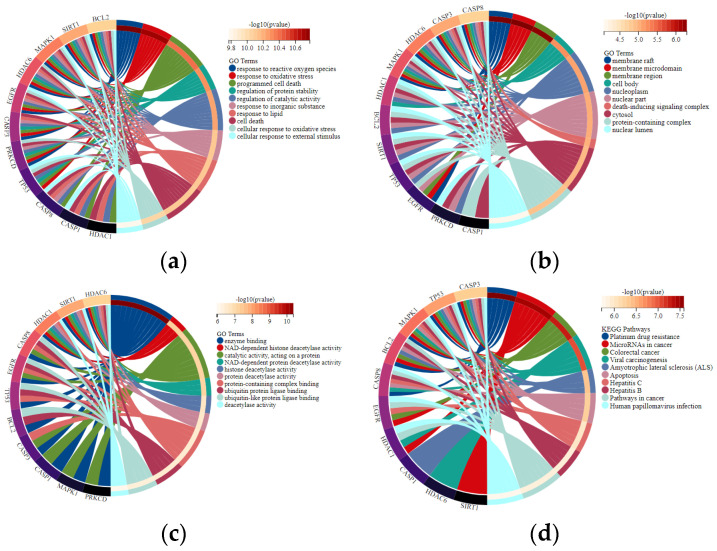
GO enrichment and KEGG pathway analysis represented in a chord plot: (**a**) biological processes; (**b**) cellular components; (**c**) molecular functions; (**d**) signaling pathways.

**Figure 5:**
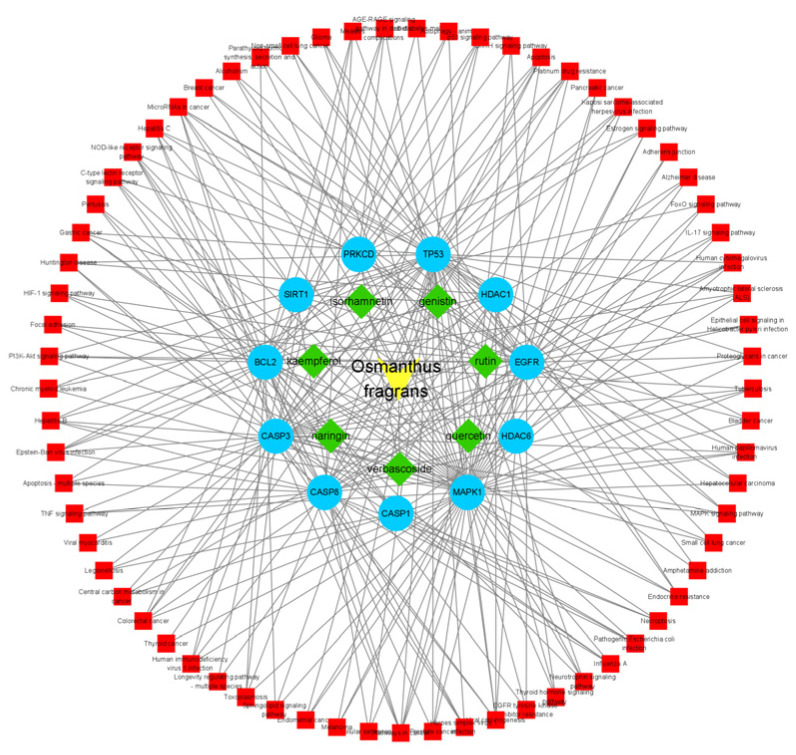
Component–target–pathway. Green: 7 main components of osmanthus extract; blue: 11 overlapping genes; red: all KEGG signaling pathways.

GO enrichment analysis revealed that 11 overlapping genes are involved in multiple biological processes (Figure 4A), including response to drugs, external stimulus and regulation of apoptotic process, cellular protein metabolic process, and signal transduction. Concerning cellular components (Figure 4B), the overlapping genes were enriched in the nucleoplasm, nuclear lumen, and endomembrane system. With regards to molecular functions (Figure 4C), they were primarily associated with enzyme binding, protein-containing complex binding, catalytic activity, and ubiquitin–protein ligase binding. Pathways enriched by KEGG were closely related to microRNAs in cancer, apoptosis, and the NOD-like receptor signaling pathway (Figure 4D). The overlapping targets and KEGG pathways of the osmanthus fragrans extract, autophagy, and osteogenesis were constructed as a network map, as shown in Figure 5. The network map contains the 7 main components of the osmanthus fragrans extract, 11 overlapping targets, and all KEGG signaling pathways.

### 2.4. Molecular Docking

**Figure 6:**
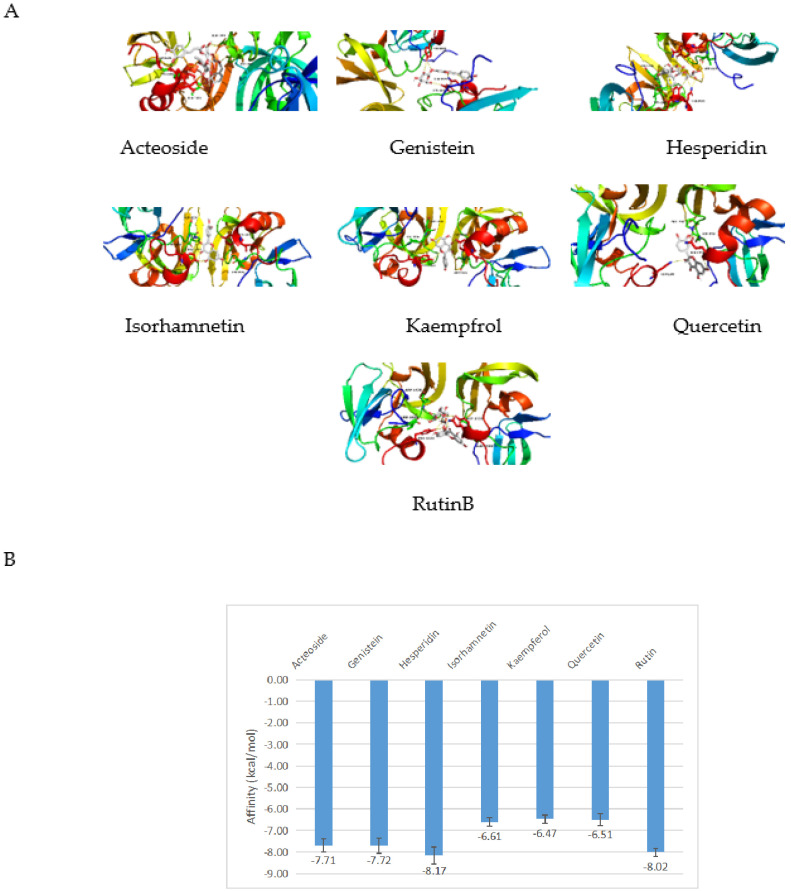
Molecular docking models of osmanthus fragrans extract and TP53: (**A**) 7 main components of osmanthus fragrans extract were docked with TP53; (**B**) the affinity of each component with TP53.

The molecular docking model of the osmanthus fragrans extract and the key target TP53 molecule was constructed, and the CDOCKEREN-ERGY value was calculated (Figure 6). It is generally believed that the lower the binding energy of the ligand to the receptor, the more stable the binding conformation. The binding energy is less than −4.25 kcal/mol, which indicates that the ligand has a certain binding activity with the receptor: less than −5.0 kcal/mol has better binding activity, and less than −7.0 kcal/mol has strong binding activity [17]. The results showed that the binding energies of each component of the osmanthus fragrans extract with TP53 were less than −5.0 kcal/mol, and especially the binding energies of acteoside, genistein, hesperidin, and rutin were less than −7.0 kcal/mol, suggesting that the osmanthus fragrans extract had good binding activity to the potential key target TP53.

### 2.5. UV-Vis Absorption Spectrum of Osmanthus Extract

**Figure 7:**
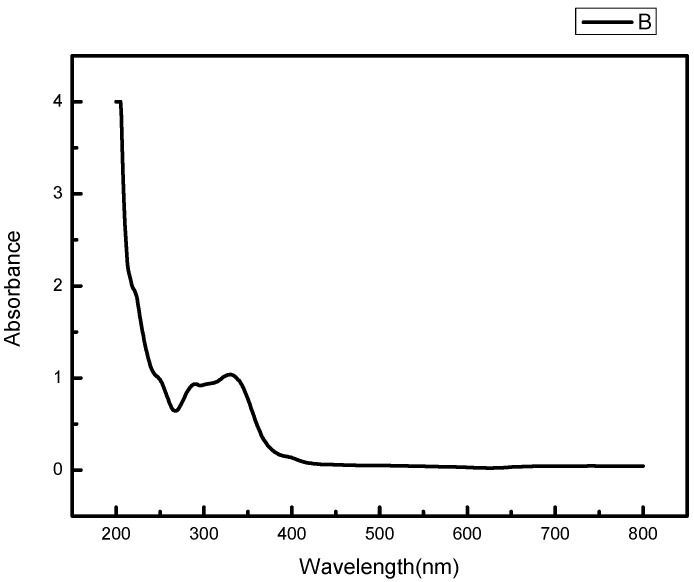
UV-Vis absorption spectrum of osmanthus extract.

The UV-visible absorption spectrum of osmanthus extract has two strong absorption peaks in the range of 200–400 nm, and the absorption wavelengths are 267 nm and 330 nm respectively, which have the typical UV spectral characteristics of flavonoids (Figure 7). The absorption peak in the range of 300–400 nm is mainly produced by the B-ring cinnamoyl system, and the absorption peak in the 240–285 nm range is produced by the A-ring benzoyl system [18].

### 2.6. XRD Pattern of OF/NIPAAM Hydrogels

**Figure 8:**
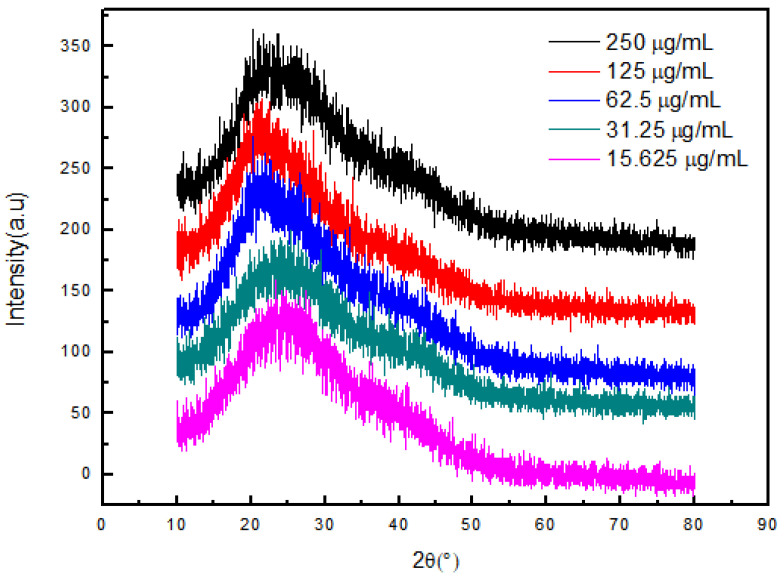
X-ray diffraction patterns of OF/NIPAAM hydrogels with different concentrations of osmanthus fragrans extract.

Figure 8 shows the X-ray diffraction patterns of NIPAAM, the osmanthus fragrans extract, and OF/NIPAAM hydrogels, respectively. It can be seen that the characteristic peak position of NIPAAM appears at 2θ = 19.7° [19] and the osmanthus fragrans extract has broad diffraction peaks at 15–30°. After NIPAAM was cross-linked with the osmanthus fragrans extract, the characteristic peak of the NIPAAM prepolymer was covered by the diffraction peak of the osmanthus fragrans extract, and no new characteristic peak appeared in the OF/NIPAAM hydrogels. In addition, the characteristic peaks of the osmanthus fragrans extract can be seen in OF/NIPAAM, which proves that the osmanthus fragrans extract was successfully mixed into the hydrogel. Moreover, with the increase in the impurity content of the osmanthus fragrans extract, the intensity of characteristic peaks of the osmanthus fragrans extract in the hydrogel increased sequentially.

### 2.7. Effect of Extract on the Cell Viability of MC3T3-E1 Cells

**Figure 9:**
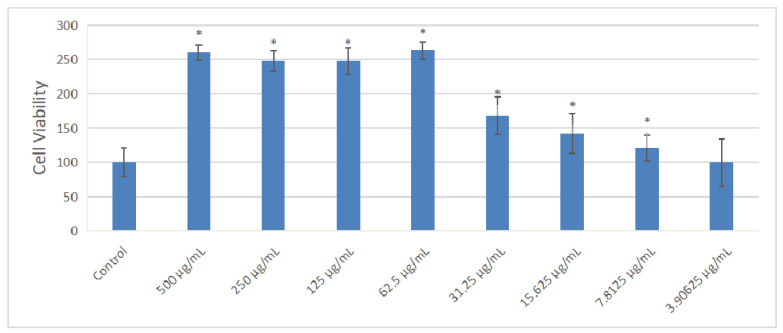
Effect of osmanthus fragrans extract on cell viability of MC3T3-E1. Different concentrations of osmanthus extract were co-cultured with MC3T3-E1 cells for 72 h, and cell viability was detected by CCK-8 assay: *, *p* < 0.05 compared with control group.

The effect of OF/NIPAAM at different concentrations (500, 250, 125, 62.5, 31.25, 15.625, 7.8125, and 3.90625 μg/mL) of osmanthus fragrans on the proliferation of MC3T3-E1 cells was detected by CCK-8 assay after 72 h incubation. Compared with the control group, as shown in the Figure 9, the osmanthus fragrans extract significantly increased the proliferation, especially at concentrations greater than or equal to 62.5 μg/mL (*p* < 0.05). There was no significant difference in the promotion of the osmanthus fragrans extract at the concentration between 62.5 and 500 μg/mL, while the concentration between 62.5 and 3.90625 μg/mL had a concentration-dependent effect. The higher the concentration, the stronger the promotion. 

### 2.8. Osmanthus Fragrans Extract Increases ALP Activity in Osteoblasts

**Figure 10:**
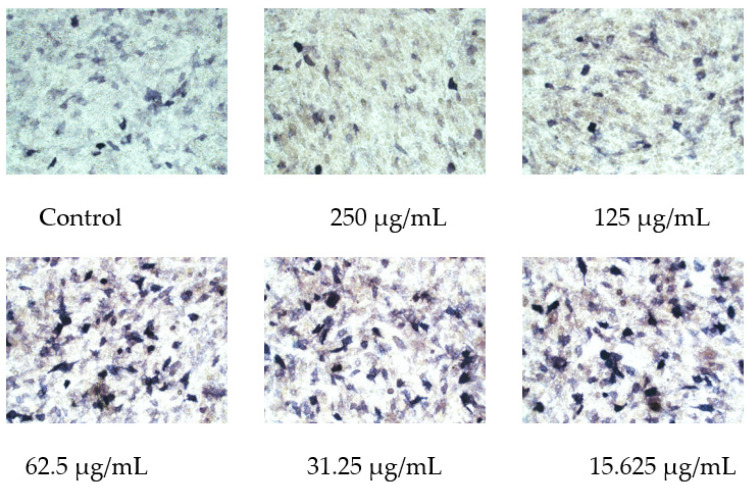
Effect of osmanthus fragrans extract on ALP staining of MC3T3-E1. ALP staining was performed after MC3T3-E1 cells were treated with different concentrations of osmanthus fragrans extract for 7 days. The coloration observed under the microscope (×20).

After MC3T3-E1 cells were treated with OF/NIPAAM hydrogels with different concentrations of the osmanthus fragrans extract for 7 days, ALP staining was performed. The range and depth of staining represent the magnitude of ALP activity. In the groups with concentrations equal to or less than 62.5 μg/mL, the range of shade was larger, in the form of clumps and darker staining, while the group with a concentration higher than 62.5 μg/mL had the lighter and more sparse coloration, similar to the control group (Figure 10).

### 2.9. Osmanthus Fragrans Extract Encourages the Formation of Mineralized Nodule

**Figure 11:**
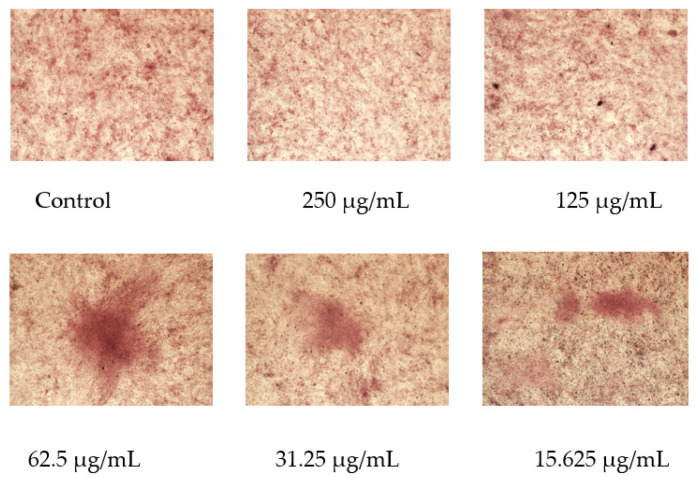
Effect of osmanthus fragrans extract on alizarin red staining of MC3T3-E1. Alizarin red staining was performed after MC3T3-E1 cells were treated with different concentrations of osmanthus fragrans extract for 21 days. The mineralized nodule was observed under the microscope (×10).

After 21 days of OF/NIPAAM treatment, mineralized nodules formed in each group with alizarin red staining, among which the group at a concentration equal to or lower than 62.5 μg/mL had a more obvious promoting effect on the mineralization of MC3T3-E1 cells, and the number of formed mineralized nodules was significantly higher than that of the control group and the higher concentration groups (Figure 11).

### 2.10. Osmanthus Fragrans Extract Promotes Osteogenic Differentiation Dependent on Autophagy Upregulation

**Figure 12:**
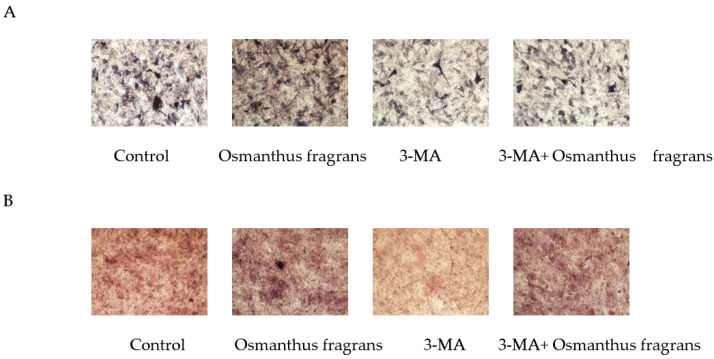
The 3-MA attenuates the osteogenic-differentiation-promoting effect of osmanthus fragrans extract: (**A**) ALP staining; (**B**) alizarin red staining.

To investigate whether the promotion of osteogenic differentiation of osmanthus extracts depends on autophagy, the autophagy inhibitor 3-MA was used, and in the subsequent ALP staining and alizarin red staining, the concentration of the osmanthus fragrans extract was selected as 62.5 μg/mL. Results showed that the addition of 3-MA attenuated ALP activity and the formation of mineralized nodules of the osmanthus fragrans extract (Figure 12).

## 3. Discussion

Oxidative stress and autophagy play important roles in maintaining bone metabolism homeostasis. Oxidative stress is an independent risk factor, which can inhibit osteoblast differentiation and promote its apoptosis, induce the proliferation and differentiation of osteoclast precursor cells, and increase their activity, thereby leading to bone-related diseases, especially osteoporosis [20]. The antioxidant biological activity of polyphenols has been widely used in various clinical fields, such as anti-cancer [21], lowering blood lipids [22], preventing atherosclerosis [23] and cardiovascular diseases [24], etc. However, little research has been conducted on its role in osteogenesis.

To investigate whether osmanthus fragrans extracts, which contain various polyphenols, have the effect of promoting bone formation and whether the mechanism involves autophagy, a network pharmacology approach was used. A total of 11 overlapping genes of osmanthus fragrans related, osteogenesis-related, and autophagy-related genes were obtained. The top three proteins by degree value are TP53, CASP3, and SIRT1. Caspase-3 (CASP3), a well-known player in apoptosis and inflammation, was recently shown to have a non-apoptotic function during osteogenesis [25]. As for SIRT1, it was reported to play a resistive role in H_2_O_2_-induced oxidative stress during osteogenesis [26]. TP53 is an important tumor suppressor gene in cells. The encoded tumor protein p53 acts as a “guardian” of genome stability and normal cellular physiological processes, and plays a key role in regulating the differentiation, proliferation, and recoding of various types of stem cells [27]. Over-expressed tumor protein 53-induced nuclear protein 2 (TP53INP2), one of the proapoptotic target genes of p53, promotes osteogenic differentiation by activating Wnt/β-catenin signaling in vitro [28]. In order to further explore the role of TP53 in the osmanthus fragrans extract in promoting osteogenic differentiation, seven components of the extract were molecularly docked with TP53. The results showed that all seven components had strong binding force to TP53, indicating that osmanthus extracts may play a pro-osteogenic effect through it. 

In order to verify the osteo-promoting effect, the osmanthus fragrans extract and OF/NIPAAM hydrogels were prepared by the ethanol reflux method and electron beam radiation, respectively. The results of UV-Vis spectroscopy showed that the main components of the extract were polyphenolic compounds, which was consistent with the literature search [18,29], while the XRD results indicated that the osmanthus fragrans extract was successfully incorporated into the NIPAAM prepolymer. Next, CCK-8, ALP staining, and alizarin red staining were used to verify the effect of OF/NIPAAM hydrogels on promoting osteogenic differentiation in vitro. The results of CCK-8 showed that extracts with concentrations above 7.8125 μg/mL promoted the proliferation of MC3T3-E1 cells. The characteristics of osteogenic differentiation are the secretion of ALP and the formation of mineralized nodules in vitro. ALP activity is an important phenotypic marker in the early stage of osteoblasts, which directly reflects the activity of osteoblasts, and alizarin red staining is mainly used to evaluate the degree of mineralization in the later stages of osteoblast differentiation. According to the characteristics, ALP staining and alizarin red staining were usually used to compare the level of osteogenic differentiation. After ALP staining, cells in the groups with concentrations equal to or less than 62.5 μg/mL showed positive expression, and blue ALP-positive granules could be seen in the cytoplasm, some of which could be fused into sheets. Alizarin red staining was also used in this experiment. Consistent with ALP staining results, there were more mineralized nodules in the groups with the concentrations lower than 62.5 μg/mL, and the staining was orange-red, with irregular shapes and different sizes. 

Autophagy is the fundamental mechanism for maintaining the viability of every cell and maintains the homeostasis of the intracellular environment by recycling damaged organelles, faulty proteins, invading pathogens, and other toxic cytoplasmic components through the lysosomal pathway. Studies have shown that during osteogenic differentiation, autophagic activity is activated, and inhibition of autophagy inhibits osteogenic differentiation [30]. In order to clarify whether autophagy is involved in osmanthus fragrans extracts in promoting osteogenic differentiation of MC3T3-E1 cells, 3-MA, a pharmacological blocker of the PI3K kinase, was used to inhibit autophagy in subsequent experiments. The experimental results showed that the addition of 3-MA down-regulated ALP activity and reduced the formation of mineralized nodules of OF/NIPAAM hydrogels. It suggested that inhibiting autophagy restrained the enhancement of OF/NIPAAM hydrogels on the osteogenic differentiation of MC3T3-E1 cells. 

## 4. Conclusions

First, the potential targets and mechanism of an osmanthus fragrans extract in regulating osteogenic differentiation based on autophagy were analyzed by network pharmacology and molecular docking. Then, osmanthus fragrans was extracted using the ethanol reflux method and an OF/NIPAAM hydrogel was prepared by electron beam radiation. The chemical components of the osmanthus fragrans extract and the microstructure of the OF/NIPAAM hydrogels were characterized by ultraviolet-visible spectrophotometry (UV-Vis) and X-ray diffraction (XRD), respectively. The cell proliferation activity, alkaline phosphatase (ALP,) and mineralized nodule formation on MC3T3-E1 with the OF/NIPAAM hydrogel were also studied to confirm its ability to promote osteogenic differentiation and explore its mechanism based on autophagy. There is a very important scientific significance for OF/NIPAAM hydrogels for osteogenesis to enrich and complement the application of hydrogel in the field of natural drug delivery.

## 5. Materials and Methods

### 5.1. Data Collection 

We searched for keywords such as “Osmanthus fragrans”, “main components” in CNKI database and Wanfang database for data mining. The Smiles chemical formula for each component was downloaded from the Pubchem database (https://pubchem.ncbi.nlm.nih.gov, accessed on 20 August 2022) and uploaded to SwissTargetPrediction (http://www.swisstargetprediction.ch/index.php, accessed on 20 August 2022) platform to screen the target genes. The keyword “osteogenesis” was entered in the GeneCards database (https://www.genecards.org, accessed on 20 August 2022) to obtain osteogenesis-related targets. The autophagy-related genes were obtained from Human Autophagy Database (http://www.autophagy.lu/index.html, accessed on 20 August 2022). 

### 5.2. Overlapping Gene Identification and Protein–Protein Interaction (PPI) Network Construction 

The screened osmanthus fragrans, osteogenesis-, and autophagy-related genes were overlapped by Venny 2.1 (http://bioinfogp.cnb.csic.es/tools/venny/, accessed on 20 August 2022). The PPI information of osmanthus fragrans–osteogenesis (drug–disease) overlapping genes and osmanthus fragrans–osteogenesis–antophagy (drug–disease–mechanism) overlapping genes was constructed by the STRING database (https://string-db.org/, accessed on 20 August 2022) and visualized by Cytoscape software (version 3.6.1, Cytoscape Consortium, San Diego, CA, USA). 

### 5.3. Gene Ontology (GO) Enrichment and Kyoto Encyclopedia of Genes and Genomes (KEGG) Pathway Analysis

Osmanthus fragrans–osteogenesis–antophagy (drug–disease–mechanism) overlapping genes were performed GO enrichment (including biological process, cellular component, and molecular function) and KEGG pathway analysis using the Database for Annotation, Visualization, and Integrated Discovery (DAVID) (https://david.ncifcrf.gov/, accessed on 20 August 2022). The main biological functions and pharmacological mechanisms of overlapping genes were screened using a cutoff of *p* < 0.05. The generated results were represented in a chord plot using Sangerbox 3.0 (Hangzhou Mugu Technology Co., LTD, Hangzhou, China) clinical bioinformatics analysis platform. The main signal pathways obtained from KEGG enrichment and the overlapping targets of osmanthus fragrans–osteogenesis–antophagy were imported into Cytoscape 3.6.1 (Cytoscape Consortium, USA) to construct and visualize the “component–target–pathway” interaction network relationship.

### 5.4. Molecular Docking Verification

The 2D structure of osmanthus fragrans extract was downloaded from the Pubchem database (http://zinc.docking.org/, accessed on 10 September 2022), and the 3D structure of the overlapping targets from the PDB database. PyMol software was used to delete water molecules and small molecule ligands of the protein structure, and AutoDockTooIs was imported for pretreatment such as hydrogenation. Finally, the extract components were docked with the target protein, and the average binding energy data were calculated as the result of molecular docking.

### 5.5. Preparation of Osmanthus Ethanol Extract and Detection of Components

Osmanthus fragrans was extracted using the ethanol reflux method according to the following extraction conditions: 80% ethanol was added at the ratio of material to liquid 1∶40 (g/mL), the reflux extraction time was 3 h, and the extraction temperature was 90 °C. The obtained extract was concentrated by rotary evaporation of ethanol at 65 °C in a rotary evaporator and filtered through a 0.45 μm microporous membrane to obtain osmanthus fragrans extract [31]. The absorption spectrum of osmanthus fragrans extract was tested by UV-Vis spectrophotometer, and the wavelength range was 200–800 nm.

### 5.6. Preparation of OF/NIPAAM Hydrogel 

A certain amount of NIPAAM was added with deionized water to prepare a 10% NIPAAM solution, and the monomer was completely dissolved by ultrasonic treatment. Different concentrations of osmanthus extract were added and vacuum-packed and sealed under anaerobic conditions. Electron beam irradiation was performed at room temperature using a 1 MeV electron beam accelerator with a total irradiation dose of 25 kGy and a dose rate of 5 kGy/pass. The hydrogel samples were dried and pulverized with a universal pulverizer to obtain fine powders. An X-ray diffractometer was used to irradiate the samples to observe the microstructure of hydrogels.

### 5.7. Cell Culture and CCK-8 Assay

Mouse pre-osteoblasts MC3T3-E1 were cultured in α-MEM medium containing 10% fetal bovine serum, 100 U/mL penicillin and streptomycin, and placed in an incubator with 5% CO_2_ and 37 °C. MC3T3-E1 cells at a density of 1 × 10^4^/well were seeded in a 96-well plate, with 3 replicate wells in each group. When the cells were confluent to about 80%, the old medium was discarded and replaced with osteogenic induction medium, and then different concentrations of OF/NIPAAM hydrogel were added for induction stimulation. After 72 h, the proliferation ability of cells was detected by CCK-8 kit.

### 5.8. ALP Staining

The cells were seeded in a 24-well plate at a density of 2 × 10^4^ cells/well, and after the cells reached 60% to 70% confluence, they were replaced with osteogenic induction media containing different concentrations of OF/NIPAAM hydrogel. After 7 days of induction, cells were fixed in 4% paraformaldehyde for 20 min at room temperature. Then, 500 μL of freshly prepared ALP incubation solution was added and incubated at room temperature in the dark. After 30 min, the samples were observed and photographed under an inverted microscope.

### 5.9. Alizarin Red Staining 

To determine osteogenic differentiation related to mineralized nodule formation, calcification deposits of cells cultured with OF/NIPAAM hydrogel at 21 days were assessed by alizarin red staining. Briefly, after osteogenic incubation, cells in 6-well plates were fixed in 4% paraformaldehyde for 10 min at room temperature and washed three times carefully. Subsequently, plates were stained with fresh 2% alizarin red liquid at pH 4.2 for 30 min in the dark to wait for dye to bind calcium salt selectively. The unbound stain and excess dye were removed gently with tap water. Samples were air-dried and stained calcium nodes were visualized and photographed under the optical microscopy.
